# Current State of Canine *Heartworm* in Portugal

**DOI:** 10.3390/ani14091300

**Published:** 2024-04-25

**Authors:** Joana Esteves-Guimarães, Jorge Isidoro Matos, Beatriz Leal-Sousa, Pedro Oliveira, Luís Lobo, Ana Cristina Silvestre-Ferreira, Carla S. Soares, Iván Rodríguez-Escolar, Elena Carretón, Rodrigo Morchón, Ana Patrícia Fontes-Sousa, José Alberto Montoya-Alonso

**Affiliations:** 1 Clínica Veterinária Aanifeira, 4520-409 Santa Maria da Feira, Portugal; joana.eg@gmail.com; 2 Internal Medicine, Faculty of Veterinary Medicine, Research Institute of Biomedical and Health Sciences (IUIBS), University of Las Palmas de Gran Canaria, 35413 Las Palmas de Gran Canaria, Spain; jorge.matos@ulpgc.es (J.I.M.); elena.carreton@ulpgc.es (E.C.); rmorgar@usal.es (R.M.); alberto.montoya@ulpgc.es (J.A.M.-A.); 3 Clínica dos Gatos, 4100-207 Porto, Portugal; beatrizlsbryant@gmail.com; 4 EPIUnit, ICBAS—Abel Salazar Institute of Biomedical Sciences, University of Porto, 4050-313 Porto, Portugal; pnoliveira@icbas.up.pt; 5 Veterinary Hospital of Porto, 4250-475 Porto, Portugal; luis.lobo@onevetgroup.pt; 6 Faculty of Veterinary Medicine, Lusófona University, 1749-024 Lisboa, Portugal; 7 Centro de Estudos de Ciência Animal (CECA), Instituto de Ciências, Tecnologias e Agroambiente (ICETA) da Universidade do Porto, Praça Gomes Teixeira, Apartado 55142, 4051-401 Porto, Portugal; 8 Center for Animal and Veterinary Science (CECAV), Associate Laboratory for Animal and Veterinary Science (AL4AnimalS), Department of Veterinary Sciences, University of Trás-os-Montes and Alto Douro (UTAD), 5000-801 Vila Real, Portugal; aferreir@utad.pt (A.C.S.-F.); carlasoares.medvet@gmail.com (C.S.S.); 9 VetLamaçães, Veterinary Clinic, 4715-303 Braga, Portugal; 10 CIVG—Vasco da Gama Research Center, EUVG—Vasco da Gama University School, 3020-210 Coimbra, Portugal; 11 Zoonotic Diseases and One Health GIR, Biomedical Research Institute of Salamanca (IBSAL), Faculty of Pharmacy, University of Salamanca, Campus Miguel Unamuno, 37007 Salamanca, Spain; ivanrodriguez@usal.es; 12 Department of Immuno-Physiology and Pharmacology, Center for Pharmacological Research and Drug Innovation (MedInUP), Veterinary Hospital of the University of Porto (UPVET), ICBAS—Abel Salazar Institute of Biomedical Sciences, University of Porto, 4050-313 Porto, Portugal

**Keywords:** *Dirofilaria immitis*, dogs, Portugal, epidemiology, districts, antigen tests

## Abstract

**Simple Summary:**

Portugal’s favourable climate renders it a hotspot for *Dirofilaria immitis* in dogs, leading to endemicity. This study aimed to provide an updated assessment of disease prevalence in Portuguese dogs, considering various factors. A total of 1367 dogs were tested, revealing an overall prevalence of 5.9%. The disease is spreading northward, with coastal areas exhibiting higher rates. Aveiro has experienced a significant increase, while the prevalence in other regions has either stabilised or decreased. Outdoor activities and older age were identified as risk factors for infection. No cases were found in the Azores. The study highlights the need for preventive measures and public awareness to combat this zoonotic disease.

**Abstract:**

The favourable geo-climatic conditions in Portugal have made it highly conducive to the development of *Dirofilaria immitis* in dogs, leading to its identification as an endemic region. This nematode is rapidly spreading across Europe, particularly in northeastern countries. The objective of this study was to provide an updated assessment of the prevalence of this disease in Portuguese dogs, analysing the results in relation to epidemiological and geo-environmental factors, and to identify potential risk factors. A total of 1367 dogs from all continental and insular districts were included in the study and tested for *D. immitis* antigens. The overall prevalence was found to be 5.9%. It was observed that the disease is spreading northward, with previously unaffected districts now reporting cases, and that the prevalence in coastal districts exceeded that of inland ones. Notably, the Aveiro district exhibited a significant increase in *D. immitis* prevalence, while in certain districts such as Setúbal, Santarém, Madeira, or Faro, a stabilisation or decrease in prevalence was noted. Furthermore, outdoor and older dogs were found to be at a higher risk of infection. No positive cases were detected in the Azores. Most of the infected dogs were located in areas of high and medium risk of infection. This study underscores the importance of implementing pharmacological prophylaxis, vector control strategies, and public awareness programs to control the spread of this zoonotic disease.

## 1. Introduction

Dirofilariosis is a vector-borne zoonotic disease caused by the parasite *Dirofilaria immitis*. Over 60 species of mosquitoes are susceptible to infection with *D. immitis*, with those of the *Anopheles*, *Aedes*, and *Culex* genera considered major vectors [1,2]. Following transmission by mosquitoes, larvae undergo development into the adult stage within carnivores [2]. These adult parasites migrate to the caudal pulmonary vascular tree, often reaching the main pulmonary arteries and the right heart in cases of massive parasitic burdens [2]. Consequently, this disease is commonly referred to as heartworm disease (HWD). Both wild and domestic canids serve as definitive hosts, exhibiting a lengthy asymptomatic period during which they act as significant reservoirs. However, endothelial damage caused by the worms, along with the release of the endosymbiotic bacterium *Wolbachia pipientis*, leads to vascular and pulmonary inflammation, pulmonary hypertension, fibrosis, and the sudden death of adult worms, potentially resulting in right heart failure [2,3].

In feline patients, most inoculated worms do not mature. However, even with a low burden, cats are highly immunologically susceptible to this infection and often exhibit clinical signs quite distinct from those observed in dogs. These signs may include chronic cough, laboured breathing, or even sudden death, resulting from the massive inflammatory response to the death of the worms, including immature ones [2,3]. Humans serve as accidental hosts for *D. immitis*, and its incidence is increasing [4,5,6,7,8,9]. In humans, dirofilariosis usually presents as pulmonary, forming nodules that resemble malignancy [5,7,10], though it can also migrate to subcutaneous tissues [8,11], the parietal pleura [12], eye [13], or liver [14]. Thus, dirofilariosis represents a significant global public health concern.

Southern European countries such as Portugal, Spain, and Italy are considered endemic regions [15,16,17,18], but dirofilariosis is increasingly spreading in northeastern countries, with reports in both veterinary and human specimens [19,20,21,22]. International trade and transportation facilitate the movement of microfilaremic reservoirs and pets traveling from regions free of *D. immitis* to endemic zones, often without chemoprophylaxis. Climate trends also play a role, facilitating the establishment of new invasive mosquito species and promoting optimal reproduction conditions of local ones. As a result, regions such as Portugal are highly conducive to the spread of this disease [23]. The evolving trends in global warming are creating favourable conditions in Europe for the spread of invasive mosquito species, such as *Ae. aegypti* and *Ae. albopictus*, both of which are capable vectors for *D. immitis* [24,25]. *Ae. albopictus* was initially identified in Portugal in 2018 and has since established itself and spread across the country, with reports from both the northern and southern regions [26,27,28,29]. While it is currently only present on Madeira Island [23,30] and has not yet reached mainland Portugal, predictive models indicate an increased risk of *Ae. aegypti* spreading globally if global warming continues unchecked [31]. Furthermore, the urbanisation of areas adjacent to water bodies (such as rivers, lakes, marshlands, and irrigated crops), as well as the creation of microclimates in urban areas leading to the formation of heat islands, support the accelerated development of *D. immitis* larvae in vectors, even during colder months. These factors collectively contribute to the clear trend of disease expansion observed over the last decade [22].

Despite some localised and regional studies, it has been over a decade since the last nationwide report on dogs was published. To our knowledge, this is the first study to assess each district individually. The objective of this research was to provide an updated overview of the prevalence of *D. immitis* in domestic dogs across each district of mainland and insular Portugal. Additionally, we aimed to examine correlations with geographic, climatic, and other epidemiological factors, as well as explore potential risk factors.

## 2. Materials and Methods

### 2.1. Location and Climatology

Portugal is situated in the southern region of Western Europe, with its continental part occupying the western section of the Iberian Peninsula. The archipelago of the Azores is in the middle of the northern Atlantic Ocean, positioned between continental Portugal and the United States of America. The Madeira archipelago is situated in the northeastern Atlantic Ocean, approximately 400 km north of the Canary Islands. Portugal spans an area of 92,090 km^2^, with a coastal extension of 1230 km in its continental part, 667 km in the Azores, and 250 km in Madeira. The country boasts unique hydrographic formations, such as Aveiro’s Ria, a 75 km^2^ marshland teeming with diverse animal and plant species. Additionally, there is the Ria Formosa estuary, spanning 60 km and characterised by a labyrinth of canals, islands, marshlands, and sandy beaches in the Algarve region. Furthermore, Portugal is home to the Tejo estuary, one of Europe’s most significant wetlands.

**Figure 1:**
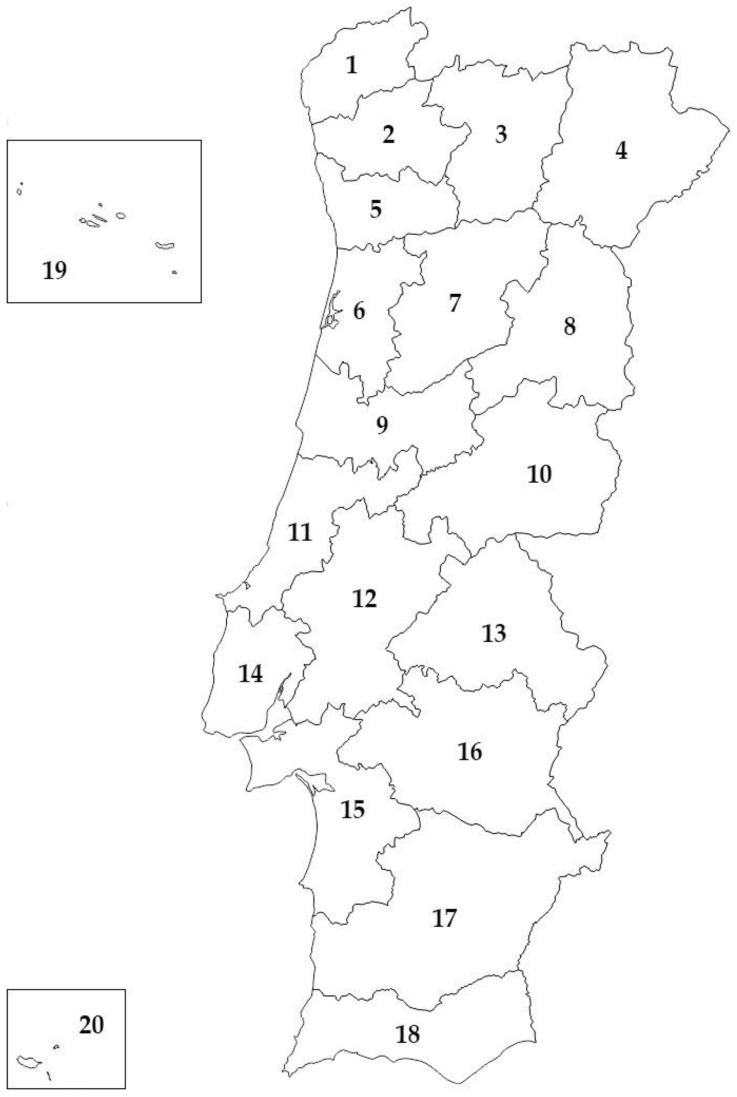
Districts of Portugal, continental and insular, included in this study. The numbers correspond to the districts listed in Table 1.

Portugal is divided into 20 districts, including those encompassing the islands (Figure 1 and Table 1). According to the nomenclature of territorial units for statistics, NUTS2 subdivisions, Portugal is further categorised into seven geographical regions: North, Centre, Lisbon Metropolitan Area, Alentejo, Algarve, and the Autonomous Regions of Madeira and Azores. The North region comprises the districts of Viana do Castelo, Braga, Bragança, Vila Real, Porto, and certain municipalities from the Aveiro, Viseu, and Guarda districts. The Centre region encompasses the remaining municipalities from the Aveiro, Viseu, and Guarda districts, along with the districts of Castelo Branco, Coimbra, and Leiria, as well as some municipalities from the Santarém and Lisbon districts. The Lisbon Metropolitan Area includes selected municipalities from the Lisbon and Setúbal districts, while Alentejo comprises the remaining municipalities from Santarém, Setúbal, and Lisbon, in addition to the districts of Évora, Beja, and Portalegre. The Algarve region corresponds to the Faro district, while the Autonomous Regions of Azores and Madeira encompass their respective island municipalities. Viana do Castelo, Braga, Porto, Aveiro, Coimbra, Leiria, Lisbon, Setúbal, and Faro are classified as coastal districts, while Vila Real, Bragança, Viseu, Guarda, Castelo Branco, Santarém, Portalegre, Beja, and Évora are designated as inland districts. The Autonomous Regions of Madeira and Azores are classified as the districts of Madeira and Azores, respectively.

On the mainland, the predominant climate according to the KÖppen classification is temperate, with average temperatures during the coldest months ranging between 0 and 18 °C [32]. The subtype Csa climate (temperate with dry or hot summers, with mean temperatures in the warmest month of >22 °C) prevails in the Iberian Peninsula, covering most of the central and southern regions of Portugal (including the districts of Beja, Castelo Branco, Évora, Faro, Lisbon, Portalegre, Santarém, and Setúbal). The temperate subtype climate Csb (temperate with dry or temperate summers, with an average temperature in the hottest month below or equal to 22 °C, and with four months or more with average temperatures above 10 °C) encompasses almost the entire west coast of Portugal and its northern regions (including the districts of Aveiro, Braga, Bragança, Coimbra, Guarda, Leiria, Porto, Viana do Castelo, Vila Real, Viseu, and the Autonomous Region of Madeira) [32,33]. In the Azores archipelago, the predominant climate is temperate with no dry season and a mild summer (Cfb), with other subtypes occurring in specific locations on some islands. For instance, Pico Island has a temperate climate with no dry season and a short, cool summer climate (Cfc) in a narrow band around Mount Pico, as well as a polar climate subtype tundra (ET), where the average temperature during the warmest month is above 0 °C, observed on Mount Pico [33]. In the Madeira archipelago, the predominant climate is temperate (Csb), although a dry climate is observed in almost all of Porto Santo Island (Bsh, hot steppe) [33].

### 2.2. Samples and Assays

The study included 1367 blood samples randomly collected from domestic dogs presented for consultation at 48 veterinary clinics and hospitals, located in 1 of the 20 districts of Portugal, between 2016 and 2023. The participation of the veterinary centres was voluntary, while dog owners were informed and provided consent for their dogs to participate in the study. Inclusion criteria for dogs included being over 6 months of age, having no prior history of *D. immitis* infection, and not receiving regular chemoprophylaxis. Additionally, epidemiological data such as age, sex, weight, length of fur, habitat, city, and postcode were recorded.

Blood samples were collected from either the cephalic or jugular vein and subjected to testing for the detection of *D. immitis* antigens (Ags), not related with the female genital apparatus, detecting both male and female parasites, by using an immunochromatography technique (Uranotest^®^ Dirofilaria, Uranovet, Barcelona, Spain), following the manufacturer’s instructions. In brief, 20 μL of whole blood, serum, or plasma was added, along with two drops of reagent, to each of the test strips. The sensitivity of the tests, as declared by the manufacturer, was 94%, with a specificity of 100% (compared to necropsy).

### 2.3. Dirofilaria Immtis Risk Map and Its Validation

To obtain a *D. immitis* risk map for Portugal, we used the methodology previously described by Rodríguez-Escolar et al. [34]. In fact, we performed a final habitat suitability model for *Cx. pipiens* in Portugal in the Iberian Peninsula with 19 bioclimatic variables and 5 environmental variables with the KUENM package in R (1.1.10). Then, we produced a number-of-generations map for *D. immitis* in R-4.3.0 software. Finally, we multiplied the final habitat suitability model for *Cx. pipiens* and the number-of-generations map for *D. immitis* using the ArcMap 10.8 raster calculator (ESRI, 2020, Redlands, CA, USA). Particular symbols were added to facilitate the interpretation of the map.

For the validation of the *D. immitis* risk map, we georeferenced points of *D. immitis*-infected dogs in all the districts of Portugal in the Iberian Peninsula and superimposed them onto the risk map to see in which area they inhabited. These maps were not available for the Azores and Madeira archipelagos due to a lack of information regarding the vectors.

### 2.4. Statistical Analysis

Data were analysed using SPSS Base 25.0 software (SPSS Inc./IBM, Chicago, IL, USA). A descriptive analysis of the qualitative variables was carried out considering the number of cases and percentages. Chi-square and Fisher exact tests to compare proportions were performed. Age, sex, fur length, weight, lifestyle, climate, geographical area, and the presence of *D. immitis* were considered as variables in the analysis. The significance level was established at *p* < 0.05. 

## 3. Results

**Figure 2:**
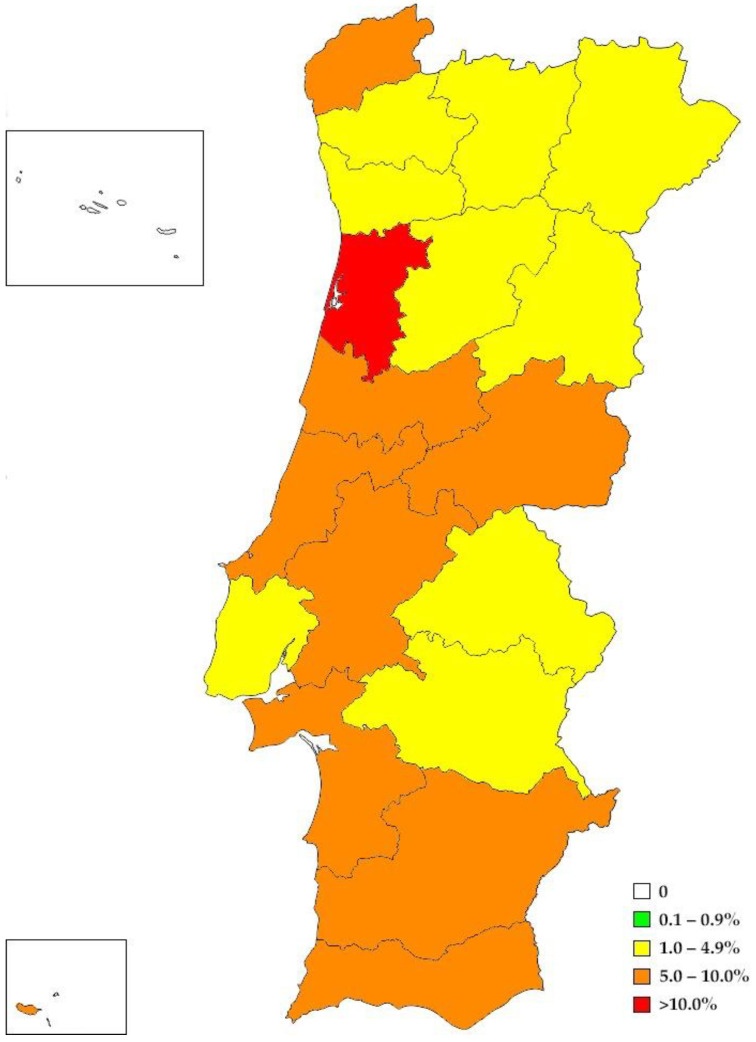
Prevalence map for *D. immitis* in domestic dogs in continental and insular Portugal by districts. In the Aveiro district (depicted in red), the white area represents Aveiro’s Ria.

The overall prevalence of circulating *D. immitis* antigens in domestic dogs from Portugal was 5.9%. Table 1 and Figure 2 display the results obtained by districts. The districts with the highest prevalence included Aveiro (15.0%), Coimbra (9.9%), Beja (9.7%), and Faro (8.8%), followed by Setúbal (8.7%), Castelo Branco (7.4%), and Viana do Castelo (6.3%). All these districts exhibited a prevalence higher than the national average. Conversely, the lowest prevalence was observed in Vila Real (1.4%), Porto (2.9%), Guarda (3.1%), and Bragança (3.3%). The Azores was the only region where no dogs tested positive for the *D. immitis* antigen.

**Figure 3:**
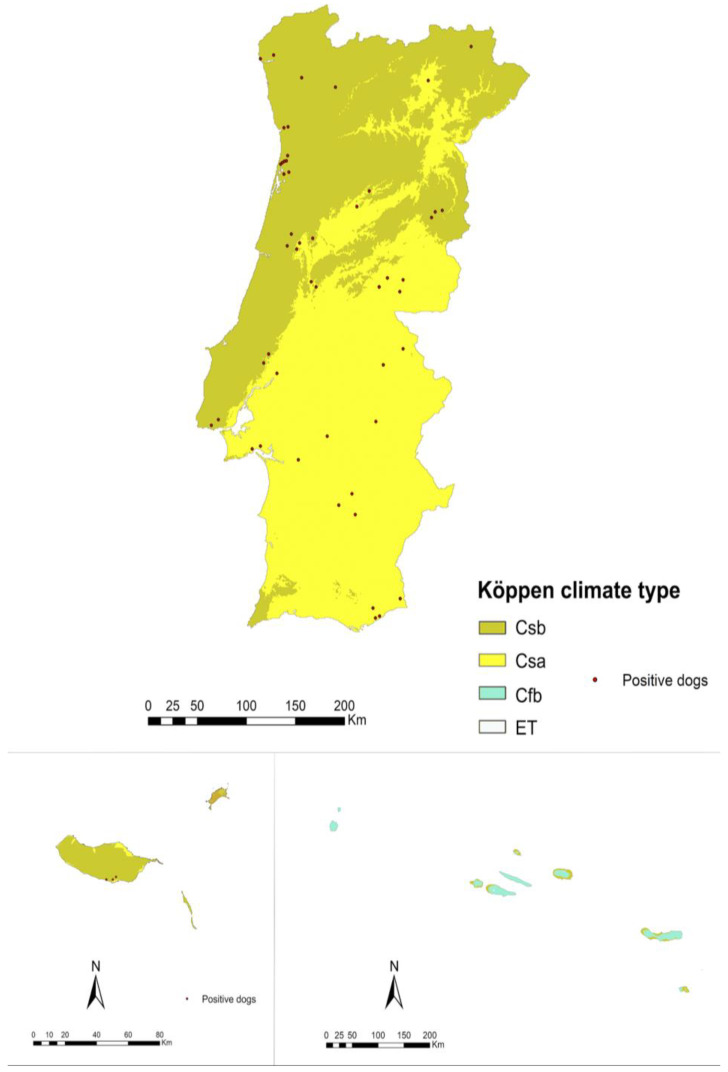
Location of different climates (Köppen Climate Classification System) in continental and insular Portugal, and geolocation of the dogs infected by *D. immitis* (dots). Legend: Csa: hot-summer Mediterranean climate; Csb: warm-summer Mediterranean climate; Cfb: temperate oceanic climate; ET: tundra climate.

The distribution of various climates across Portugal and the location of positive dogs can be observed in Figure 3. The statistical analysis revealed no significant differences between climates, although the lowest prevalence was consistently observed in districts with a Csb climate classification. Additionally, the Azores exhibited a prevalence of zero, representing the only district characterised by a Cfb climate.

Table 2 presents the results related to sex, age, and geographical area. Of the tested dogs, 53% were male and 47% were female. While no statistically significant differences were observed between males and females, males exhibited a slightly higher prevalence (6.6%) compared to females (5.0%). The sampled dogs were further categorised by age into the following groups: <1 year (3.8%), 1–4 years (35.3%), 5–10 years (45.6%), and >10 years (15.3%). Although no statistically significant differences were found between age groups, dogs aged 5–10 years demonstrated a higher prevalence (8.2%) compared to other age groups (0.0%, 4.0%, and 4.8%).

No significant differences in prevalence were observed according to geographic area (*p* > 0.05). The prevalence in the Azores was noted to be zero (0.0%), while it was higher along the coastal regions (7.4%), followed by Madeira (5.8%) and inland areas (4.7%). A prevalence among dogs over 10 years of age was higher in Madeira and along the coast compared to that in inland areas, as well as among dogs aged 1 to 4 years. However, there were no differences observed in prevalence based on sex and geographic area (*p* > 0.05).

In terms of lifestyle, 2.7% of the sampled animals were stray dogs, 9.7% were strictly kept indoors, 55.5% were kept indoors but had regular access outdoors, and 32% were kept exclusively outdoors. Among the positive cases, 62.4% were outdoor dogs. Regarding coat length, 56.0% of the sampled dogs had short fur, 35.6% had medium fur, and 8.4% had long fur. Among the positive cases, 60.8% were short-haired dogs.

Of all the variables studied (age, sex, fur length, weight, lifestyle, and geographical area), age and lifestyle were identified as statistically significant risk factors (Table 3). The risk of testing positive for the *D. immitis* antigen was 5.483 times higher in outdoor dogs compared to that in indoor dogs, and 2.021 times higher in dogs older than 5 years compared to that in younger ones.

**Figure 4:**
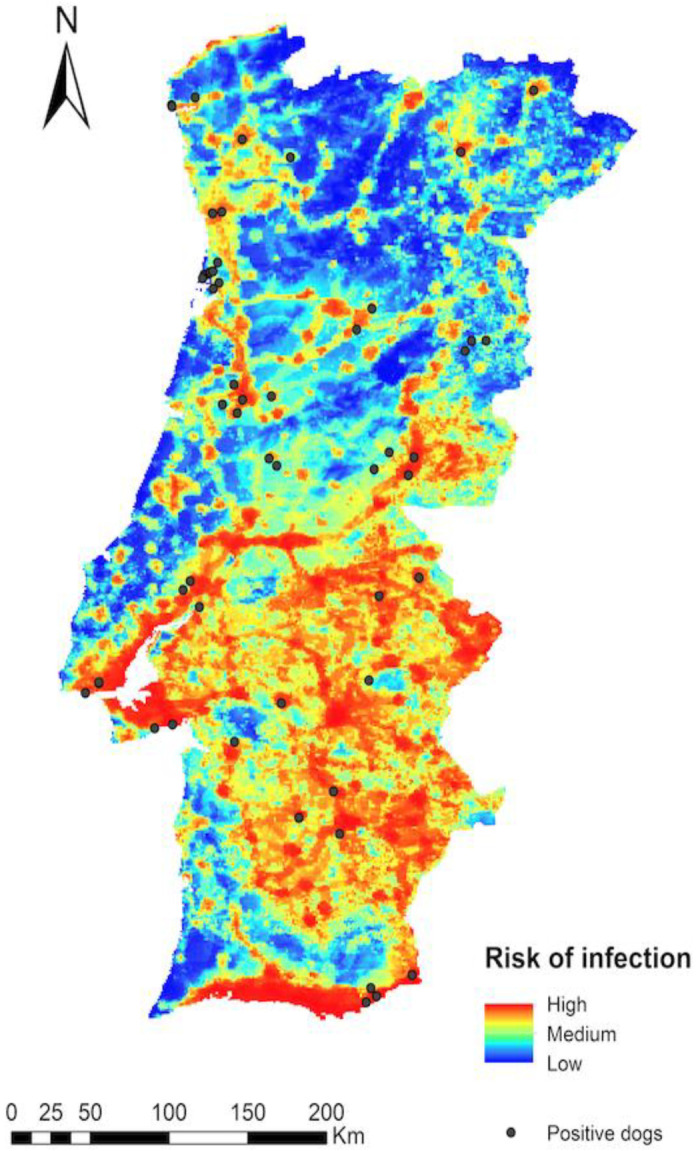
*Dirofilaria immitis* infection risk map with geolocation of the infected dogs (identified with dots) in continental Portugal.

In relation to the infection risk map for *D. immitis* in the Iberian Peninsula (Portugal) (Figure 4), when positive dogs were geo-referenced on the map, it was observed that 29.6% were in a high-risk zone, 47.9% were in a medium-risk zone, and 22.5% in a low-risk zone. It should be noted that the dogs were distributed throughout the whole peninsular area and that they were located in areas where the human footprint was high, as well as in irrigated areas and areas with natural or artificial stagnant water.

## 4. Discussion

Since 1996, the presence of *D. immitis* in Portugal has been identified [35] and investigated, with many studies being locally restricted [36,37,38,39,40]. The present study not only confirmed the establishment of dirofilariosis in dogs, but also defined areas with increasing prevalence, as well as its spread to previously unaffected regions.

In the northern region of Portugal, since the last study on privately owned dogs conducted about ten years ago, there has been a noticeable increase in HWD prevalence in the Viana do Castelo district, rising from 2.1% in 2015 [37] to the current 6.3%. Furthermore, our findings indicated that districts previously considered free of the disease, such as Bragança, Vila Real, Porto, and Braga, now exhibit prevalence rates ranging from 1.4% to 3.4%. Similar results were recently reported in northern Spain [41]. Previous data concerning other species aligns with our conclusions that *D. immitis* is spreading northward. Seroprevalence in privately owned cats ranged from 7.1% in Braga to 14.3% in Viana do Castelo [37], while in humans, it varied from 3.9% in Braga to 7.1% in Vila Real’s district [42]. Higher prevalence rates were observed in kennel dogs from the Caminha municipality (Viana do Castelo district) (48.4%) [43] and red foxes from the Peneda-Gerês National Park (Viana do Castelo, Braga, and Vila Real districts) (15.8%) [43]. 

These results were likely influenced by a combination of factors, including a lack of awareness among veterinary clinicians and pet owners regarding the presence of this parasite in northern Portugal, leading to an insufficient implementation of chemoprophylactic measures. Additionally, the emergence of invasive species, such as *A. albopictus*, first detected in the north of Portugal in 2018 [26], along with the ongoing expansion of indigenous vectors, has contributed to this trend. Predictive models suggested that by 2080, the territory occupied by *Cx. pipiens*, a known vector, is expected to increase by 50% in the Iberian Peninsula [34]. Despite being areas with less favourable temperature conditions (colder), they are also characterised by an abundance of water bodies, such as rivers and irrigated crops, which are conducive to the development of *D. immitis* vectors [44]. Moreover, the reservoir effect of kennel dogs and wildlife in the Peneda-Gerês National Park, where parasitic control measures are deficient, cannot be overlooked in these districts.

In the central region of the country, since its initial description in the Coimbra district, the prevalence of *D. immitis* in dogs has shown fluctuations. It decreased from 13.8% [40] to 8.8% [37], and in our study, it has slightly increased to 9.9%. However, in a very specific coastal area within this district, Figueira da Foz, the prevalence was significantly higher at 27.3% [36]. Just north of this area, in Dunas de Mira, *D. immitis* has also been detected in red foxes [45]. Aveiro’s district, with geospatial characteristics like those of Figueira da Foz, has experienced a notable increase in canine HWD prevalence from 6.8% in 2015 [37] to the current rate of 15.0%. Reports on feline seroprevalence in this district corroborate these findings [37], as do reports on human cases [42]. Interestingly, based on the current distribution map of *Ae. Albopictus*, it is observed that in Portugal, this vector is highly prevalent in the district of Aveiro [23].

Both the Aveiro and Coimbra districts boast significant water bodies and marshland areas, such as Aveiro’s Ria, which provide ideal conditions for vector development, as previously noted [44]. Additionally, the presence of HWD in wildlife and a lack of awareness among clinicians regarding its prevention in domestic canines and felines in these districts may have contributed to the observed expansion. Furthermore, in line with this trend, our study marked the first confirmation of canine HWD in the Guarda, Castelo Branco, and Leiria districts in central Portugal. In Leiria, our findings were supported by human data, with a case report of a pulmonary lesion initially suspected to be malignant, which was later identified as a *D. immitis* nodule [7]. In the Viseu district, previously considered free of the disease [37], the prevalence was 3.8%. Given the limited knowledge of this disease in these districts, and considering that many are secondary residences for both national and emigrant families, it is conceivable that the spread of the disease is linked to the unrestricted movement of pets from areas with a higher prevalence, subsequently acting as reservoirs for vectors present throughout the national territory [34,46,47].

In Lisbon, a previous study reported a *D. immitis* prevalence of 2.4% in apparently healthy dogs and 5.8% in clinically suspected dogs in 2012 [48], whereas our findings indicated a prevalence of 3.6%. Similar to findings in Madrid [49] and Barcelona [50], the identification of positive cases and evidence of stable-spreading HWD in urban areas is not uncommon. Lisbon, being both a coastal city and encompassing part of the Tejo estuary, characterised by marshlands, experiences mean temperatures above 14 °C for most of the year, with an extended estimated transmission period for *D. immitis* [51]. Additionally, there may be a contribution from the formation of heat islands during colder months [49,50].

To the best of our knowledge, this was the first description of the presence of canine HWD in the districts of Évora, Portalegre, and Beja. Previous data from 2012 categorised results by NUTS region, with the Alentejo region showing a prevalence of 4.7% in apparently healthy dogs and 14.0% in clinically suspected ones [48]. Our results ranged from 3.9% in Portalegre to 9.7% in Beja. In the districts of Setúbal and Santarém, since 2014, canine HWD prevalence has decreased from 24.8% to 8.7% and from 13.2% to 5.1%, respectively [40]. This decrease is likely due to increased awareness among clinicians and pet owners and improved prophylactic measures in areas that have been extensively studied [38,40,46,47,52].

Similarly, in the Algarve region, despite favourable conditions for the expansion of this parasite, such as the climate and establishment of the invasive mosquito species *Ae. albopictus* between 2018 [27] and 2020 [29], and the presence of *D. immitis* in *Culex* spp. in this area [53], the prevalence of canine HWD has decreased slightly from 9.4% in 2015 [39] to the current 8.8%. This decrease may be attributed to increased awareness of the disease. The detection of a dog infected with *D. repens* in 2016 [54], previously considered the most well-known zoonotic filaroid [55], and the description of the first infection with *D. immitis* in pinnipeds in an oceanographic park in the Algarve in 2017 [56], may have contributed to this heightened awareness.

In 2012, Madeira Island was identified as a hyperendemic area for *D. immitis*, with a prevalence of 40.0% in apparently healthy dogs [48]. Unique climatic characteristics, such as coastal temperatures consistently above 13 °C throughout the year, coupled with high humidity, provide optimal conditions for the development of mosquito populations. A recent survey in domestic cats revealed a prevalence of 3.5% [57] and our results supported a significant decrease in canine prevalence (5.8%). This reduction could be attributed to evidence of vector population control measures implemented in the area. Since 2006, *D. immitis* has been found in *C. theileri* mosquitoes on Madeira Island [58], and in the same year, *A. aegypti* was identified for the first time in the region [59]. In 2012, Cardoso and colleagues’ findings [48] coincided with a dengue outbreak that prompted highly effective vector control measures, resulting in a tenfold reduction in the *Ae. aegypti* population [30]. These measures may have not only targeted *Ae. aegypti* but also other potential vectors of HWD. Additionally, with the awareness of such a high prevalence, preventive pharmacological strategies might have been established in domestic animals, which could have aided in controlling the spread of this disease. Risk maps have not been created for Madeira Island, but given its similarities to certain parts of the Canary Islands, we might anticipate similar developments in the future. This could involve the suppressed expansion of mosquitoes into higher or more arid zones, while their establishment remains in areas with sufficient humidity, such as urban areas, irrigated crops, or areas influenced by bodies of water such as the ocean [60].

Although not statistically significant when comparing results by Köppen climates, the Azores stood out as the only district with zero prevalence, characterised by a Cfb climate. Recent data from Spain revealed similar findings in exclusively Cfb areas such as Asturias, Cantabria, and the Basque Country, where the prevalence was relatively low (<2%) [41]. To date, there have been no reported cases of HWD in the Azores. However, it should be noted that districts with some of the highest prevalence rates (such as Aveiro, Coimbra, Setúbal, and Faro) are areas with significant tourist activity, which may contribute to the further spread of the disease both nationally and internationally.

Not surprisingly, more than half of the positive dogs had short fur. A shorter coat length may increase the exposed skin area for vectors to feed. Additionally, approximately 60% of the positive dogs were strictly outdoor animals. Spending more time outdoors increases their contact with vectors, amplifying the probability of infection. However, this finding also underscores the risk for the remaining 40% of dogs that live strictly or mostly indoors, emphasizing the importance of providing similar protection for indoor dogs. Lifestyle was identified as a statistically significant risk factor, with outdoor dogs being more than 5 times as likely to be infected by *D. immitis* compared to indoor dogs. Age was also found to be a significant factor, with dogs older than 5 years being approximately 2 times more at risk of infection. This increased risk may be attributed to spending more time in potential contact with vectors that could be infected.

When comparing the positive animals based on their geographical location, we observed that most of the positive dogs were located in coastal districts and in highly populated inland districts, where the risk of *Dirofilaria* spp. infection was high in many of the cases. This difference may be attributed to various environmental and bioclimatic factors. According to Rodríguez-Escolar and colleagues [34], human activity, temperature, and humidity (affected by natural water bodies or artificial irrigated crops) are the most significant factors influencing the suitability of habitat for the reproduction of *Cx. pipiens* mosquitoes.

It is widely acknowledged that Portugal’s population is concentrated in major urban centres, with migration from the interior to coastal cities over the past decades. Despite some regions in the northern districts being colder (and less favourable), much of the coastal zone is characterised by a significant presence of water bodies, including rivers, estuaries, and marshlands, which, with their tendency to create stagnant waters, provide optimal conditions for mosquito vectors. The contribution of heat islands in some coastal cities should not be underestimated, particularly during colder months. Furthermore, the main high-altitude mountains are in interior districts, coinciding with the lowest prevalence rates. Our findings indicated that most of the positive animals were situated in high-risk zones according to these prediction models [34].

When selecting the diagnostic method for this study, our goal was to identify a test with the best combination of sensitivity and specificity, while also being rapid and practical for clinical use, given that our study involved dogs seen in private practices located within busy veterinary centres. With the detection of *D. immitis* antigens, the presence of immune complexes binding to antigens and potential test errors could lead to false negative results. Also, despite its high specificity, cross-reactions in antigen testing may occur between *D. immitis* and other species (e.g., *D. repens*, *Angiostrongylus vasorum*, or *Spirocerca lupi*) [61]. These occurrences could be reduced by employing additional diagnostic tests, such as the evaluation of microfilariae, which was not conducted in the present study and could be regarded as a potential limitation.

## 5. Conclusions

Dirofilariosis is a disease that is spreading across Europe, moving from formerly known endemic areas in the south to those in the north. This spread is attributed to a combination of factors including global warming, which leads to the proliferation of indigenous vectors and the introduction of new invasive species, as well as insufficient measures to control the disease due to lack of knowledge or other socio-economic factors. This study not only confirmed the presence of *D. immitis* in previously identified districts but also revealed the expansion of this disease to almost all of the Portuguese territory, delineating medium- to high-risk zones.

It is not only essential to control climate changes and their implications, but it is also crucial to enhance detection schemes, raise awareness, and implement prophylactic measures for domestic animals, along with strategies to control *D. immitis* vectors, to limit the spread of this disease. Lastly, while not undermining its significance, the expansion of this infection, although mostly asymptomatic in humans, also underscores the potential spread of other dangerous infections that share the same vectors.

## Figures and Tables

**Table 1:** Prevalence for *D. immitis* in domestic dogs from Portugal, by districts, climates (Köppen Climate Classification System), and geographical area. Abbreviations: *n* = number of sampled dogs; + = number of positive dogs; % = percentage of positive dogs. Legend: Csa: hot-summer Mediterranean climate; Csb: warm-summer Mediterranean climate; Cfb: temperate oceanic climate.

District	Climate	Geographical Area	*n*	+	%
**Overall**			**1367**	**80**	**5.9**
1. Viana do Castelo	Csb	Coastal	63	4	6.3
2. Braga	Csb	Coastal	59	2	3.4
3. Vila Real	Csb	Inland	72	1	1.4
4. Bragança	Csb	Inland	60	2	3.3
5. Porto	Csb	Coastal	68	2	2.9
6. Aveiro	Csb	Coastal	80	12	15.0
7. Viseu	Csb	Inland	52	2	3.8
8. Guarda	Csb	Inland	96	3	3.1
9. Coimbra	Csb	Coastal	81	8	9.9
10. Castelo Branco	Csa	Inland	81	6	7.4
11. Leiria	Csb	Coastal	37	2	5.4
12. Santarém	Csa	Inland	59	3	5.1
13. Portalegre	Csa	Inland	77	3	3.9
14. Lisbon	Csa	Coastal	83	3	3.6
15. Setúbal	Csa	Coastal	69	6	8.7
16. Évora	Csa	Inland	63	3	4.8
17. Beja	Csa	Inland	72	7	9.7
18. Faro	Csa	Coastal	80	7	8.8
19. Azores	Cfb	Azores	46	0	0.0
20. Madeira	Csb	Madeira	69	4	5.8

**Table 2:** Prevalence for *D. immitis* in domestic dogs from Portugal by sex, age, and geographical area. Abbreviations: *n* = number of sampled dogs; + = number of positive dogs; % = percentage of positive dogs.

		Coastal			Inland			Madeira			Azores			Total			*p*-Value Chi^2^
		n	+	%	n	+	%	n	+	%	n	+	%	n	+	%	
Sex	Female	273	19	7.0	296	10	3.4	42	3	7.1	31	0	0.0	642	32	5.0	0.124
	Male	347	27	7.8	336	20	6.0	27	1	3.7	15	0	0.0	725	48	6.6	0.193
Age	<1 year	19	0	0.0	27	0	0.0	3	0	0.0	3	0	0.0	52	0	0.0	-------
	1–4 years	221	13	5.9	230	5	2.2	19	1	5.3	13	0	0.0	483	19	4.0	0.185
	5–10 years	277	25	9.0	285	24	8.5	38	2	5.3	23	0	0.0	623	51	8.2	0.431
	>10 years	103	8	7.8	90	1	1.1	9	1	11.1	7	0	0.0	209	10	4.8	0.125

**Table 3:** Analysis of statistically significant risk factors of the variables studied (age, sex, fur length, and lifestyle). *n* = number of sampled dogs; % = percentage of positive or negative dogs. (*) = significant differences between the groups analysed.

		Test Result						*p*-Value Chi^2^
		Total		Negative		Positive		
		n	%	*n*	%	*n*	%	
Sex	Total	1367	100.0%	1287	100.0%	80	100.0%	0.198
	Female	642	47.0%	610	47.4%	32	40.0%	
	Male	725	53.0%	677	52.6%	48	60.0%	
Fur length	Total	1367	100.0%	1287	100.0%	80	100.0%	0.510
	Short fur	766	56.0%	718	55.8%	48	60.8%	
	Long fur	115	8.4%	111	8.6%	4	5.1%	
	Medium fur	486	35.6%	458	35.6%	28	34.2%	
Lifestyle	Total	1367	100.0%	1287	100.0%	80	100.0%	0.000 *
	Stray dogs	37	2.7%	37	2.9%	0	0.0%	
	Indoor	133	9.8%	130	10.0%	3	3.8%	
	Indoor + Outdoor	759	55.5%	732	56.9%	27	33.8%	
	Outdoor	438	32.0%	388	30.2%	50	62.4%	
Age	Total	1367	100.0%	1287	100.0%	80	100.0%	0.004 *
	<5 years	533	39.0%	514	39.9%	19	23.8%	
	≥5 years	834	61.0%	773	60.1%	61	76.2%	

## Data Availability

The data presented in this study are available on request from the corresponding author.
